# The draft genome of mandrill (*Mandrillus sphinx*): An Old World monkey

**DOI:** 10.1038/s41598-020-59110-3

**Published:** 2020-02-12

**Authors:** Ye Yin, Ting Yang, Huan Liu, Ziheng Huang, Yaolei Zhang, Yue Song, Wenliang Wang, Xuanmin Guang, Sunil Kumar Sahu, Karsten Kristiansen

**Affiliations:** 1 Laboratory of Genomics and Molecular Biomedicine, Department of Biology, University of Copenhagen, Copenhagen, 2100 Denmark; 2 BGI-Shenzhen, Shenzhen, 518083 China; 3 China National GeneBank, BGI-Shenzhen, Shenzhen, 518120 China; 4 Department of Biotechnology and Biomedicine, Technical University of Denmark, 2800 Kgs., Lyngby, Denmark; 5 State Key Laboratory of Agricultural Genomics, BGI-Shenzhen, Shenzhen, 518083 China

**Keywords:** Evolutionary genetics, Sequencing

## Abstract

Mandrill (*Mandrillus sphinx*) is a primate species, which belongs to the Old World monkey (*Cercopithecidae*) family. It is closely related to human, serving as a model for human health related research. However, the genetic studies on and genomic resources of mandrill are limited, especially in comparison to other primate species. Here we produced 284 Gb data, providing 96-fold coverage (considering the estimated genome size of 2.9 Gb), to construct a reference genome for the mandrill. The assembled draft genome was 2.79 Gb with contig N50 of 20.48 Kb and scaffold N50 of 3.56 Mb. We annotated the mandrill genome to find 43.83% repeat elements, as well as 21,906 protein-coding genes. The draft genome was of good quality with 98% gene annotation coverage by Benchmarking Universal Single-Copy Orthologs (BUSCO). Based on comparative genomic analyses of  the Major Histocompatibility Complex (MHC) of the immune system in mandrill and human, we found that 17 genes in the mandrill that have been associated with disease phenotypes in human such as Lung cancer, cranial volume and asthma, barbored amino acids changing mutations. Gene family analyses revealed expansion of several genes, and several genes associated with stress environmental adaptation and innate immunity responses exhibited signatures of positive selection. In summary, we established the first draft genome of  the mandrill of value for studies on evolution and human health.

## Introduction

Since the successful accomplishment of human genome project^1^, followed by continuous reduction in the cost of genome sequencing and drammatic increase in throughput of new sequencers, magnanimous primates genomic data are becoming available for both Old World monkeys, such as chimpanzee^2^, and New World monkeys, such as marmoset (*Callithrix jaccbus*) (Table 1). Generally, the species being selected for genome sequencing must meet certain criteria including: (1) important evolutionary position within the phylogeny (for instance, chimpanzee, gibbon and orangutan); (2) biomedical relevance to human. For example, macaque and baboon were selected as they are often used to explore the genetic basis of human diseases^3^, and squirrel monkey has been a model for studies on neurobiology and infectious diseases. These genomic data resources provided deeper understanding on genome content, evolution as well as diversity. It enabled comparative analyses of human and other primates, and primates and other mammals.

**Table 1:** Published primate genome sequences. (modified based on^37^).

Common name	Species name	Bases in contigs	Contig N50	Scaffold N50	Reference
Chimpanzee	*Pan troglodytes*	2.7 Gb	15.7 kb	8.6 Mb	^38^
Chimpanzee (updated)	*P. troglodytes*	2.9 Gb	50.7 kb	8.9 Mb	^39^
Bonobo	*Pan paniscus*	2.7 Gb	67 kb	9.6 Mb	^40^
Gorilla	*Gorilla gorilla*	2.7 Gb	11.8 kb	914 kb	^41^
Gorilla (updated)	*Gorilla gorilla*	2.8 Gb	9.6 Mb	23.1 Mb	^42^
Orang-utan	*Pongo abelii*	3.1 Gb	15.5 kb	739 kb	^43^
Indian rhesus macaque	*Macaca mulatta*	2.9 Gb	25.7 kb	24.3 Mb	^44^
Indian rhesus macaque (updated)	*M. mulatta*	3.1 Gb	107.2 kb	4.2 Mb	https://www.ncbi.nlm.nih.gov/assembly/GCA_000772875.3 ^45^
Chinese rhesus macaque	*M. mulatta*	2.8 Gb	11.9 kb	891 kb	^46^
Vietnamese cynomolgus macaque	*M. fascicularis*	2.9 Gb	12.5 kb	652 kb	^46^
baboons	Papio *baboons*	2.9 Gb	149.87 kb	140.35 Mb	^47^
Aye-aye	*D. madagascarensis*	3.0 Gb	NA	13.6 kb	^48^
Vervet	*C. aethiops*	2.8 Gb	90.4 kb	81.8 Mb	^49^
Gibbon	*Nomascus leucogenys*	2.8 Gb	35.1 kb	22.7 Mb	^50^
Marmoset	*Callithrix jacchus*	2.3 Gb	29 kb	6.7 Mb	^51^
Mouse lemur	*Microcebus murinus*	2.4 Gb	210.7 kb	108.2 Mb	https://www.ncbi.nlm.nih.gov/assembly/GCF_000165445.2
Pig-tailed macaque	*Macaca nemestrina*	2.8 Gb	106.9 kb	15.2 Mb	https://www.ncbi.nlm.nih.gov/assembly/GCF_000956065.1/#/st
Sifaka	*Propithecus coquereli*	2.1 Gb	28.1 kb	5.6 Mb	https://www.ncbi.nlm.nih.gov/assembly/GCF_000956105.1/#/st
Sooty mangabey	*Cercocebus atys*	2.8 Gb	112.9 kb	12.8 Mb	https://www.ncbi.nlm.nih.gov/assembly/GCF_000955945.1/
Squirrel monkey	*Saimiri boliviensis*	2.5 Gb	38.8 kb	18.7 Mb	https://www.ncbi.nlm.nih.gov/assembly/GCF_000235385.1/#/def
Bushbaby	*Otolemur garnettii*	2.4 Gb	27.1 kb	13.9 Mb	https://www.ncbi.nlm.nih.gov/assembly/GCF_000181295.1/
Mouse lemur	*Microcebus murinus*	2.4 Gb	182.9 kb	3.7 Mb	https://www.ncbi.nlm.nih.gov/assembly/GCF_000165445.1/
Tarsier	*Tarsius syrichta*	3.4 Gb	38.2 kb	401 Mb	^52^

*Mandrillus sphinx* (henceforth referred to as Mandrill) is a primate species living in Africa. It is a relatively  ancient monkey species belonging to the Papionini tribe. Mandrills are mostly terrestrial but they are more arboreal than baboons^4^. They live in large, stable groups with the size as big as hundreds of individuals. The largest horde which has been verifiably observed contained more than 1,300 mandrills, which is the largest non-human aggregation ever documented. Mandrills have been used as experimental models for studies of human diseases, although chimpanzee and gorilla are more closely related to human^5^. Notably, mandrills are used in immune related research such as in studies of bacterial infections^6,7^, parasite infections^8,9^ and viral diseases particularly Simian immunodeficiency viral (SIVs) infection^10,11^. But until now no genomic data on mandrill has been released impeding further scientific research.

In this study, we present a high-quality draft genome sequence of the mandrill using high throughput sequencing, making it a useful resource for future comparative genomic studies and studies related to human health.

## Results

### Genome sequencing and assembly

Whole genome sequencing of mandrill yielded 426.72 Gb of raw sequence data (142× considering the estimated genome size of 2.90 Gb). After filtering, clean reads amounting to 289.55 Gb were obtained for genome assembly, with ~73× from paired-end libraries and ~26× from mate-pair libraries (Table S1). 212.84 Gb data were used for k-mer analysis, which resulted in the distribution of depth-frequency (Fig. S1), with a secondary peak at half of the major peak coverage of ~31×. The genome size of mandrill was estimated to be 2.90 Gb with notable heterozygosity. All of the clean data were used to generate the draft genome assembly, followed by gap filling. The size of the assembled genome was 2.88 Gb (covering 99.31% of the estimated genome size). The contig N50 was 20.48 kb with the longest contig being 211.02 kb, and the scaffold N50 was 3.56 Mb with the longest scaffold being 19.10 Mb. In total, 634 of the longest scaffolds made up more than 80% of the whole genome (Table 2).

**Table 2:** Summary of the mandrill genome assembly.

	Contig		Scaffold	
	Size (bp)	Number	Size (bp)	Number
N90	5,266	141,475	638,217	936
N80	9,025	101,618	1,303,160	634
N70	12,638	75,505	1,962,294	457
N60	16,336	56,061	2,730,696	332
N50	20,483	40,751	3,564,730	241
Longest	211,017	–	19,105,867	–
Total size	2,798,997,503	–	2,882,689,325	–
Total number (> = 100 bp)	–	455,069	–	215,140
Total number (> = 2 kb)	–	194,923	–	4,742

### Genome annotation

By a combination of *de novo* and homology-based methods, about 42.22% of the assembled mandrill genome were identified as transposable elements (TEs) (Table S2). The Long Interspersed Nuclear Elements (LINEs) made up less of the mandrill genome (~17%) compared to the human genome (~21%), while the Short Interspersed Nuclear Elements (SINEs) representation was similar (~12%). Notably the *Alu* elements made up similar proportion (10%~11%) **(**Table S3**)**, reflecting that *Alu* elements are conserved within the primate genomes as previously described^12^.

The gene set of the mandrill genome contains 21,906 protein-coding genes (Table S4). The average gene length was 39,087 base pair (bp) with the average intron length of 5,785 bp. A total of 21,622 (98.70%) of the predicted genes were functionally annotated (Table S5). Three types of ncRNAs were annotated in the mandrill genome, including tRNAs, rRNAs, and snRNAs. In total, 4,278 short noncoding RNA sequences were identified in the mandrill genome (Table S6).

The quality of mandrill genome and gene completeness were assessed by conducting the Benchmarking Universal Single-Copy Orthologs (BUSCO) analysis^13^. 98% of BUSCOs were completely detected in the assembled genome (2981: complete and single-copy; 170: complete and duplicated) among 3,023 tested BUSCOs. The numbers of fragmented and missing BUSCOs were 28 and 14, respectively (Table S7).

### Gene family and phylogenetic analysis

**Figure 1:**
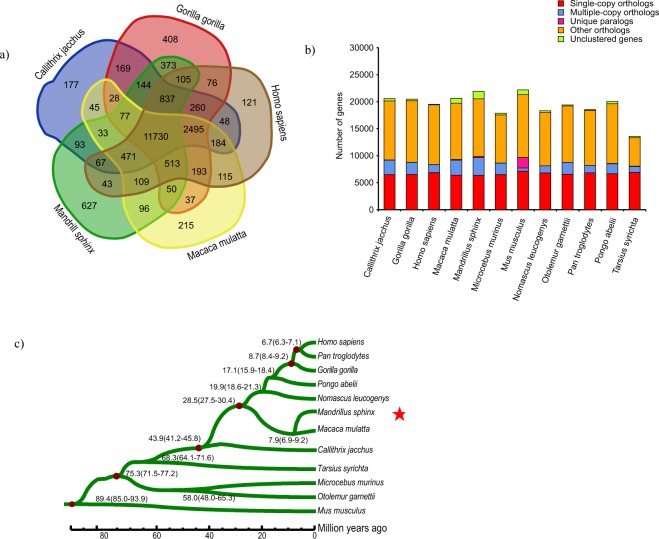
Phylogeny and evolution of the mandrill’s gene families. (**a**) The Venn diagram of the gene families of human (*Homo sapiens*), macaque (*Macaca mulatta*), gorilla (*Gorilla gorilla*), marmoset (*Callithrix jacchus*) and mandrill. (**b**) Comparison of orthologous genes among 11 primates and mouse. (**c**) The maximum-likelihood phylogenetic tree based on the 4-fold degenerate sites of 5,133 single-copy gene families in the 12 species.

For mandrill, we identified 15,368 gene families with 1,387 genes that could not be clustered among 12 species, and 87 families were found to be unique (Table S8). These unique gene families were significantly enriched in the functional annotation with GO:0006412 of translation (GO level: BP, P = 6.29e-33), and GO:0003735 of a structural constituent of ribosome (GO level, BP, P = 6.29e-33) (Table S9). Compared to human (*Homo sapiens*), macaque (*Macaca mulatta*), gorilla (*Gorilla gorilla*) and marmoset (*Callithrix jacchus*), 627 gene families, with 1,293 genes, were found to be unique in the mandrill and 5,133 single-copy orthologous genes were found to be shared among all the 12 species (Fig. 1a).

The maximum-likelihood phylogenetic tree (Fig. 1b) of the 12 species using 5,133 single-copy genes indicated that mandrill is located in the same clade as macaque and this clade diverged from the human clade about 28.5 (27.5–30.4) million years ago (MYA) while the divergence time between* Cercopithecoidea* and *Hominoidea* was estimated to be 26.66 (24.29–28.95) MYA using mitochondrial genome sequences^14^. Mandrill was estimated to split from macaque about 7.9 (6.9–9.2) MYA, which is different from the previous estimate of 6.6 (6.0–8.0) MYA^15^.

In the mandrill lineage, there were 797 expanded and 3,982 contracted gene families (Fig. S2). Expanded gene families were found to be significantly enriched in functions related to biosynthetic processes, structural constituents of ribosomes, nucleosomal DNA binding, G-protein coupled receptor activity, olfactory receptor activity, glucose catabolic process, peptidyl-prolyl isomerization, and electron transport chain pathway (Table S10). In mandrill, peptidylprolyl isomerase A (PPIA) was significantly expanded (GO:0003755, P = 3.60E-89). The PPIA belongs to the peptidyl-prolyl cis-trans isomerase (PPIase) family which catalyzes the cis-trans isomerization, folding of the newly synthesized protein, and regulates many biological processes including inflammation and apoptosis, and has even been reported to play a role in cerebral hypoxia-ischemia. In a stressed environment with the presence of reactive oxygen species, cells secrete PPIA to induce an inflammatory response and mitigate tissue injury. The peroxiredoxin-6 (PRDX6) family, which can reduce peroxides and protect against oxidative injury in relation to metabolism, was also significantly expanded (GO:0051920, P = 0.000641).

In total, 657 Positively Selected Genes (PSGs) were identified with significant enrichment in molecular functions related to kinase activity, transferase activity, and phosphotransferase activity (Table S11). The functions of these PSGs were further investigated, with 34 genes being found to be innate immunity response-related genes based on an InnateDB search. Interactions between these genes were predicted by STRING: functional protein association networks (http://string-db.org/cgi). As shown in Fig. S3, the genes *STAT1, IL5, IL1R1, ATG5, CREB1, DICER1, PIK3R1* which play important roles in the immune system, are strongly associated with stress resistance and wound healing. Besides that, PSGs related to innate immunity responses were found to be enriched in terms of GO:0080134: regulation of response to stress (GO level: BP, P = 1.59e-05), GO:0006955: immune response (GO level: BP, P = 4.11e-05) and KEGG:4640: hematopoietic cell lineage (P = 6.83e-05) (Table S12).

**Figure 2:**
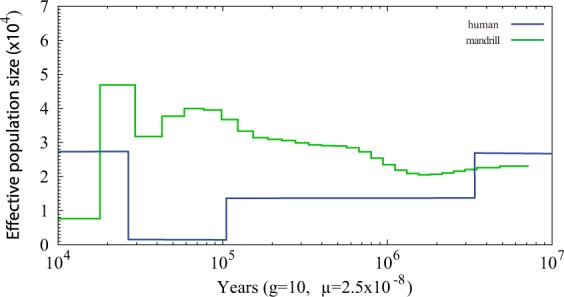
The demographic changes of mandrill compared to human. The x-axis indicates the time (from left to right indicates recent to ancient), and the y-axis indicates the estimated effective population size.

The demographic history of a species reflects historical population variation, and therefore information on the genome would be important. We inferred a noticeable population bottleneck in the demographic history of the mandrill (Fig. 2). Around 28 thousand years (kys) ago, the mandrill population went through a sharp expansion, followed by a noticeable bottleneck from a peak of 61,000 and 47,000 to ~6,500 around 17 kys ago. The expansion of the population size coincided with the increase of the human population. This might indicate that the climate change was suitable for mammal population expansion, while the recent bottleneck of mandrill populations is different from the recent increase of the human population.

### Comparative analysis of the major histocompatibility complex (MHC) region

**Figure 3:**
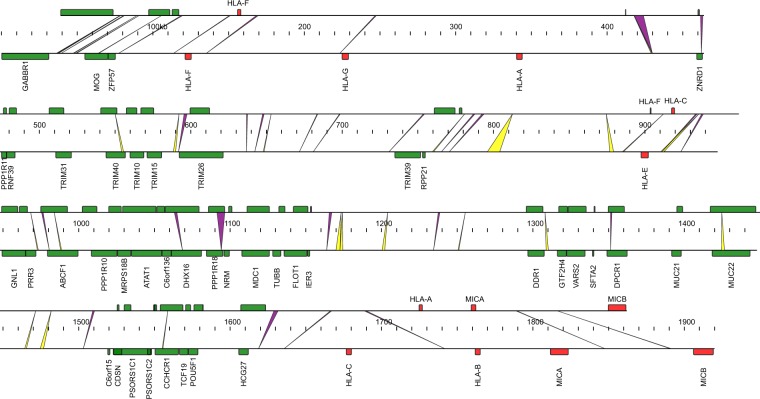
Synteny of MHC class I region between mandrill and human. Red boxes indicate the MHC genes. Green boxes indicate other protein-coding genes. Yellow and green indicate deletions and insertions in mandrill, respectively.

The MHC region harbors a series of genes to assist cells in recognizing foreign substances. Based on the assembled mandrill genomes, a relatively intact MHC region was found on chromosome 4. In order to check the assembly quality, reads that mapped back to the MHC class I region showed good coverage and pair-end/mate-pair relationships (Fig. S4), supporting proper assembly quality of the MHC region. Since the MHC region is highly repetitive, a detailed repeat annotation was carried out for both mandrill and human MHC class I regions (from gene *GABBR1* to gene *MICB* in the direction from the telomere side to the centromere side) with the same parameter to find similar repeat content for the two species in this region (48.27% in mandrill compared to 51.03% in human) (Table S13). *HLA* genes are important for immune recognition; thus, mandrill *HLA* genes were further checked and compared to human *HLAs*. In the human MHC class I region, there were 50 genes in total including six *HLAs*, while in mandrill MHC class I region, only four *HLAs* were identified. By searching the entire genome outside the MHC region, another four *HLAs* were identified, making the total number of *HLAs* to be eight in mandrill. However, further inspection of the eight *HLAs* genes in mandrill revealed that five of them harbored start or stop codon changes, or frameshift mutations resulting in premature termination (Fig. 3), reflecting possible differences in immune responses between mandrill and human.

### 3.8 Disease-related genomic features

In order to gain further insight in disease-related mutations in the mandrill, we first performed a comparative genomic analysis of human with mandrill using lastz^16^, then identifying SNPs in the mandrill. In total, 557 SNPs in the coding regions were found which were distributed among 520 genes. We collected the information related to mutations from the HGMD database, and checked the presence/absence of such mutations in the mandrill genome. Based on this analysis, we found amino acid changes in 17 genes suggesting disease-related mutation (Table 3). Moreover, we found that some of the mutations are in the functional domains, which may strongly affect the function of these genes (Table S14). These mutations have been associated with disease phenotype in humans, in relation to lung cancer, cranial volume, and asthma.

**Table 3:** Genes with disease and its’ mutation in mandrill.

Gene name	Full name	Position	Wildtype AA	Mutation AA	Disease Description
*ALAD*	Aminolevulinate Dehydratase	59	K	N	Amyotrophic lateral sclerosis
*CIITA*	Class II Major Histocompatibility Complex Transactivator	500	G	A	Multiple sclerosis
*CRB1*	Crumbs Cell Polarity Complex Component 1	959	G	S	Retinitis pigmentosa
*IL4R*	Interleukin 4 Receptor	75	I	V	Asthma, atopic
*MCPH1*	Microcephalin 1	761	A	V	Cranial volume
*NPHS2*	NPHS2 Stomatin Family Member, Podocin	192	I	V	Nephrotic syndrome
*TP53BP1*	Tumor Protein P53 Binding Protein 1	353	D	E	Lung cancer

## Discussion

Primates are well-studied mammals because of their evolutionary importance as well as their close relationship to human. Since the first publication of the gorilla genome in 2012, seven non-human primate genomes have become available and their genomic features comprehensively studied. Despite current progress in primate genomic studies, more genomic data for primate species are needed. Here, by utilizing the high throughput sequencing technologies, we established the draft genome for the mandrill, which is a valuable resource for primate and human diseases-related studies. The contig N50 of the genome was longer than 20 kb while the scaffold N50 reached 3.56 Mb, indicating good quality of genome assembly. In order to further improve the genome assembly, long reads sequencing might be applied to fill in the gaps of the assembly. Other than that, with further development of technologies like formaldehyde cross-linking and sequencing (Hi-C sequencing), the assembled scaffolds might be further anchored to the chromosomes. The repeat content and gene content in the mandrill were similar to other primate species. According to the phylogenetic tree established based on single-copy gene families, mandrill was found to diverge from the human clade about 28.5 MYA.

In our study, the genomic features of mandrill were comprehensively explored and compared with human. 797 gene families were expanded and enriched including families involved in  G-protein coupled receptor activity, olfactory receptor activity, glucose catabolic processes, peptidyl-prolyl isomerization. Furthermore, 657 PSGs were identified in mandrill, 34 of which were found to be innate immunity response genes. Some of these might be involved in rapid initiation of the innate immune responses in mandrill^17^. Several key genes including *STAT1, IL5, IL1R1, ATG5, CREB1, DICER1, PIK3R1* are know to play important roles in the immune system and strongly associated with stress resistance and wound healing.

The mandrill is commonly used as a model for humans with both species possessing characteristic features, including genetic mechanisms underpinning the immune system, language ability, as well as the olfactory system. In terms of the immune system, the MHC regions were specifically analyzed in the mandrill genome. A remarkable synteny was found between the mandrill and the human MHC region with only 54 insertions and deletions (longer than 100 bp). A substantial expansion of olfactory receptor genes was found in mandrill compared to other species, indicating a unique olfactory systems in the mandrill to be further examined.

The assembly and analysis of the draft mandrill genome also emphasize the potential in establishing more genomes for primate species. Primates are an order of mammal species with ~about16 families and ~500 species, which are all highly evolved animal species with special physiological and behavioral characteristics. Despite the evolutionary importance and relatively simple genome content, reference genomes have only been established for ~20 species, and the genome assemblies varied in quality and continuity impeding further in depth analysis and applications. Thus, establishing draft genomes by new sequencing technologies for all primate species would be invaluable for evolutionary studies, conservation/preservation, as well as human genetic/disease research and applications.

## Methods

### Sample preparation

We obtained 5 mL blood from the left jugular vein of an eighteen-year-old male mandrill from Beijing Zoo. The blood was collected in a plastic collection tube with 4% (w/v) sodium citrate, snap-frozen in liquid nitrogen and stored at −80 °C. Genomic DNA was extracted using the AXYGEN Blood and Tissue Extraction Kit according to the manufacturer’s instructions. To assess the quality, the extracted DNA was subjected to electrophoresis in 2% agarose gel and stained with ethidium bromide. The DNA concentration was detected by Quant-iT™ PicoGreen^®^ dsDNA Reagent and Kits (Thermo Fisher Scientific, USA) according to the manufacturer’s protocol.

### Ethics statement

Animal blood collection was approved by both Beijing Zoo and BGI-IRB. The study was further carried out based on the agreement between BGI and Beijing Zoo. Moreover, the utility was in accordance with guidelines from the China Council on Animal Care.

### Library establishment and sequencing

The mandrill DNA was used for library formation, following a previously published protocol^18^. A total of seven libraries were constructed, and sequencing was carried out on the Illumina sequencer HiSeq2000. Of the seven libraries, three were short insert size libraries including insert sizes of 250 bp (sequenced to 150 bp at two ends), 500 bp and 800 bp (sequenced to 100 bp at two ends), respectively. The other four libraries were mate-pair libraries with insert sizes of 2 kb, 5 kb, 10 kb and 20 kb (sequenced to 90 bp at two ends). SOAPnuke was used to filter reads according to the criterions^19^: (i) reads with more than 10% Ns (ambiguous bases); (ii) reads with more than 40% of low-quality bases (quality score less than 10); (iii) reads contaminated by adaptor (adaptor matched 50% with no more than one base mismatch) as well as PCR duplicated reads (identical reads at both ends).

### Genome assembly

In order to assess genome features, 17-mers (17 bp sub-sequences) were extracted and subjected to the K-mer analysis. Reads from 250 bp, 500 bp and 800 bp insert libraries were used for this analysis.

Then the genome was assembled by short-reads assembly software SOAPdenovo2^20^ using the filtered data (with parameter settings pregraph-K 35; contig -M 1; scaff). Gaps were filled using paired sequence data from 3 libraries (250, 500, and 800 bp) with -p 31 parameters by GapCloser.

### Transposable elements and repetitive DNA

RepeatMasker v4.0.5^21^ and Repeat-ProteinMask were used to scan the whole genome for known transposable elements in the RepBase library v20.04^22^. Then, RepeatMasker was applied again for identifying *de novo* repeats based on the custom TE library constructed by combining results of RepeatModeler v1.0.8 (RepeatModeler,RRID:SCR_015027) and LTR_FINDER v1.0.6^23^. Prediction of tandem repeats was also done using Tandem Repeat Finder v4.0.7^24^ with the following setting: Match = 2, Mismatch = 7, Delta = 7, PM = 80, PI = 10, Minscore = 50, MaxPeriod = 2000.

### Protein-coding gene and non-coding RNAs annotation

After masking known TE repeat elements, genes were predicted using three methods, including homolog based, evidence-based and *ab initio* prediction. For homolog based annotation, protein sequences of *Macaca mulatta* (Ensemble 73 release), *Pan troglodytes* (Ensemble 73 release), *Nomascus leucogenys*, *Pongo abelii* (Ensemble 73 release), *Gorilla gorilla* (Ensemble 73 release), and *Homo sapiens* (Ensemble 73 release) were aligned to the mandrill genome using BLAT^25^. Then GeneWise^26^ (Version 2.2.0) was used for further precise alignment and gene structure prediction. For ab initio prediction, we employed AUGUSTUS^27^ (Version 3.1) to predict gene models in the repeat masked genome. Finally, the gene prediction results were combined using GLEAN^28^. In order to identify the function of the final gene set, three databases (SwissProt, KEGG, and TrEMBL databases) were searched for best matches using BLASTP (version 2.2.26) with an E-value of 1e-5. The InterProScan software^29^, which searches Pfam, PRINTS, ProDom and SMART databases for known motifs and domains, was also used for the gene function annotation.

To identify tRNAs, tRNAscan^30^ was used. While for rRNAs identification, 757,441 rRNAs from the public domain were used to search against the genome with command -p blastn -e 1e-5. To identify RNA genes and other non-coding RNA (ncRNA), Rfam database^31^ was used to search against the genome.

### Gene family clustering

Protein sequences of 11 species including *Gorilla gorilla*, *Homo sapiens*, *Macaca mulatta*, *Microcebus murinus*, *Nomascus leucogenys*, *Otolemur garnettii*, *Pan troglodytes*, *Pongo abelii*, *Tarsius syricht*, *Callithrix jacchus*, and *Mus musculus* were used to cluster the gene families. TreeFam (http://www.treefam.org/) was used to defined gene families in *Mandrillus sphinx*. Firstly, all-versus-all blastp was used with the e-value cutoff of 1e-7 for 12 species and then the possible blast matches were joined together by an in-house program. Thirdly, we removed genes with aligned proportion of less than 33% and converted bit score to percent score. Finally, hcluster_sg (Version0.5.0, https://pypi.python.org/pypi/hcluster) was used to cluster genes into gene families. We selected single-copy orthologs as the set of genes that remained single copy in each species.

### Phylogenetic tree construction

With gene family clusters defined, the four-fold degenerate (4D) sites of 5,133 single-copy orthologous among the 12 species were extracted for the phylogenetic tree construction. PhyML package^32^ was used to build the phylogenetic tree with maximum-likelihood methods and GTR + gamma as an amino acid model (1,000 rapid bootstrap replicates conducted). Based on the phylogenetic tree, divergence times of these species were estimated by MCMCTree (http://abacus.gene.ucl.ac.uk/software/paml.html) with the default parameters. To further calibrate the evolution time in the tree, six fossil dates collected from the TimeTree database (http://www.timetree.org/) were used, including the divergence time between *Mus musculus* and human to be 85–93 MYA^33^, divergent time between human and chimpanzee, gorilla, to be 6 MYA (with a range of 5–7)^34^ and 9 MYA (range, 8–10)^35^.

With the gene family clustering result, contraction and expansion could be detected to investigate the dynamic evolutionary changes along the phylogenetic tree. According to the phylogenetic tree and divergence time, CAFÉ^36^ was used for gene family contraction and expansion analysis.

Demography was estimated using the pairwise sequentially Markovian coalescent (PSMC) model with the following setting: -N25 –t15 –r5 –p “4 + 25*2 + 4 + 6”. In order to scale the results to real-time, 10 years per generation and a neutral mutation rate of 2.5e-08 per generation were used.

### Gene family expansions/contractions and positively selected genes

The selection pressure in mandrill was measured by comparing nonsynonymous (dN) and synonymous (dS) substitution rates on protein-encoding genes. This ratio would be equal to 1 if the whole coding sequence evolves neutrally. When dN/dS < 1, it’s under constraint, and when dN/dS > 1 it should be under positive selection. PSGs was detected using models in the program package PAML version 3.14, neutral (M1 and M7) and selection (M2 and M8) models were used.

## Supplementary Material

Supplementary Information.

Supplementary Information 2.

Supplementary Information 3.

## Data Availability

1. NCBI Sequence Read Archive SRP134063 (https://trace.ncbi.nlm.nih.gov/Traces/sra/?study = SRP134063). 2. CNGB Nucleotide Sequence Archive CNP0000251. 3. NCBI Assembly GCA_004802615.1 (https://www.ncbi.nlm.nih.gov/assembly/GCA_004802615.1).
