# Supplementary material for: The draft genome of mandrill (Mandrillus sphinx): An Old World monkey
Source: Sci Rep. 2020 Feb 12;10:2431. doi: 10.1038/s41598-020-59110-3 (PMC7016171; doi:10.1038/s41598-020-59110-3)
Supplement: Supplementary file 1 — Supplementary Information. [file 41598_2020_59110_MOESM1_ESM.pdf]

The draft genome of mandrill (*Mandrillus sphinx*): An Old World monkey

Ye Yin[1,2*#], Ting Yang[2,4*], Huan Liu[1,2,5*], Ziheng Huang[2,3], Yaolei Zhang[2,3], Yue Song[2,3], Wenliang Wang[2,3], Xuanmin Guang[2], Sunil Kumar Sahu[2,3,5], Karsten Kristiansen[1#]

1. University of Copenhagen, Copenhagen 2100, Denmark
2. BGI-Shenzhen, Shenzhen 518083, China
3. China National GeneBank, BGI-Shenzhen, Shenzhen 518120, China
4. Department of Biotechnology and Biomedicine, Technical University of Denmark, 2800 Kgs. Lyngby, Denmark
5. State Key Laboratory of Agricultural Genomics, BGI-Shenzhen, Shenzhen 518083, China

# Correspondence should be addressed to Ye Yin (yinye@bgi.com) and to Karsten Kristiansen (kk@bio.ku.dk)

* These authors contributed equally to this work.

Table S1. Statistics of the sequencing data.

Library type	Insert size	Read length	Raw data (Gb)	Clean data (Gb)	Sequence depth (×)
Paired-end	250	150	113.296	91.18	37.77
Paired-end	500	100	83.054	67.38	27.68
Paired-end	800	100	65.328	54.28	21.78
Mate-pair	2,000	90	34.561	18.71	11.52
Mate-pair	5,000	90	32.967	16.3	10.99
Mate-pair	10,000	90	65.377	31.35	21.79
Mate-pair	20,000	90	32.141	10.34	10.71
Total	-	-	426.72	289.55	142.2

Table S2. Categories of TEs in mandrill genome.

	RepBase TEs		TE Proteins		*De novo*		Combined TEs	
	Length (bp)	%	Length (bp)	%	Length(bp)	%	Length (bp)	%
**DNA**	47,923,460	1.66	13,264,158	0.46	27,821,997	0.96	68,516,869	2.37
**LINE**	401,922,498	13.9	229,014,482	7.94	725,287,701	25.1	815,296,990	28.28
**SINE**	319,811,862	11.0	--	--	481,314,186	16.6	576,217,301	19.99
**LTR**	169,184,719	5.87	39,705,383	1.38	80,223,826	2.78	200,629,837	6.96
**Other**	81	--	--	--	3,210	0	3,291	0
**Unknown**	--	--	--	--	2,897,396	0.1	2,897,396	0.1
**Total**	936,130,281	32.4	281,888,845	9.78	1,117,858,14	38.78	121,695,029	42.22

Note: Repbase TEs, the result of *RepeatMasker* based on Repbase; TE proteins, the result of *RepeatProteinMask* based on Repbase; *De novo*, Result of *RepeatMasker* by using library predicted through *De novo* prediction; Combined: combined results of Repbase TEs, TE proteins and *de novo*.

Table S3. Repeat elements comparison between mandrill, human and mouse.

Group	Percentage in the genome		
	Mandrill	Human	Mouse

**LINE**	16.61	20.99	19.2
**L1**	15.05	17.37	18.78
**L2**	1.39	3.3	0.38
**LINE/other**	0.17	0.32	0.04
**SINE**	12.10	13.64	8.22
**Alu**	10.47	10.74	2.66
**MIR**	1.37	2.9	0.57
**B4**	0.20	--	2.36
**SINE/other**	0.06	--	2.64
**LTR**	8.36	8.55	9.87
**MaLRs**	3.40	3.78	4.82
**Other ERVs**	4.85	4.77	4.4
**LTR/other**	0.11	--	0.65
**DNA transposons**	3.27	3.03	0.88
**Other**	0.06	0.53	0.74
**Total**	40.40	46.74	38.91

Table S4. Summary of gene annotation for mandrill genome.

**Gene set**		**Number**	**Transcript length**	**CDS length**	**Exons**	**Exon length**	**Intron length**
De novo	**AUGUSTUS**	18,460	54,148	1,429	8.68	164.65	6,863
**Homolog**	***Nomascus leucogenys***	20,874	39,863	1,499	8.56	175.07	5,072
	Pongo abelii	23,330	37,371	1,373	7.82	175.53	52,757
	Pan troglodytes	20,866	40,317	1,502	8.46	177.62	5,204
	Macaca mulatta	25,460	38,089	1,294	7.36	175.96	5,787
	Gorilla gorilla	23,791	34,748	1,413	7.92	178.47	4,816
	Homo sapiens	25,161	39,338	1,513	8.42	179.77	5,098
**EST**		38,021	7,365	781	2.33	335.00	4,935
**Final set**		21,906	39,087	1,390	7.52	184.95	5,785

Table S5. Function annotation of the mandrill genes.

		Gene number	%
**Total**		21,906	100.00
**Annotated**	InterPro	18,139	82.80
	GO	14,160	64.64
	KEGG	18,022	82.27
	Swissprot	20,547	93.80
	TrEMBL	21,529	98.28
	All database	21,622	98.70
**Unannotated**		284	1.30

Table S6. Summary of non-coding RNA genes in mandrill genome

Type		Copy	Average length	Total length	% of genome
**tRNA**		466	75.36	35,118	0.12
	rRNA	982	97.05	95,301	0.33
	18S	20	252.6	5,052	0.02
	28S	205	160.49	32,902	0.11
**rRNA**	5.8S	8	103.87	831	0.00
	5S	749	75.45	56,516	0.19
	snRNA	2716	110.76	300,830	1.04
	CD-box	880	101.76	89,547	0.31
**snRNA**	HACA-box	314	136.82	42,963	0.15
	splicing	1261	118.27	149,146	0.52

Table S7. Summary statistics of BUSCO analysis.

	Mandrill
**Total BUSCO groups**	3,023
**Complete BUSCOs**	2,981
**Complete and single-copy BUSCOs**	2,811
**Complete and duplicated BUSCOs**	170
**Fragmented BUSCOs**	28
**Missing BUSCOs**	14

Table S8. Gene family clustering in the twelve species.

Species	Genes number	Un-clustered genes	Family number	Unique families	Average gene per family
Callithrix jacchus	20,585	445	16,858	12	1.19
Gorilla gorilla	20,478	313	17,495	8	1.15
Homo sapiens	19,513	105	17,367	2	1.12
Macaca mulatta	20,627	912	16,391	38	1.2
**Mandrill**	21,906	1,387	15,368	87	1.34
Microcebus murinus	17,853	310	15,414	9	1.14
Mus musculus	22,190	864	17,778	209	1.2
Nomascus leucogenys	18,312	308	16,643	2	1.08
Otolemur garnettii	19,390	180	16,667	3	1.15
Pan troglodytes	18,523	144	16,764	3	1.10
Pongo abelii	20,065	461	17,354	21	1.13
Tarsius syrichta	13,589	262	12,243	13	1.09

Note: Un-clustered genes refer to unique genes in the species; Unique families refer to unique gene families of the species.

Table S9. GO enrichment of unique gene families in mandrill.

GO ID	GO term	GO class	P value
**GO:0044260**	cellular macromolecule metabolic process	BP	2.02E-04
**GO:0043170**	macromolecule metabolic process	BP	6.18E-04
**GO:0009987**	cellular process	BP	1.32E-02
**GO:0008152**	metabolic process	BP	1.88E-02
**GO:0044238**	primary metabolic process	BP	2.69E-02
**GO:0044237**	cellular metabolic process	BP	3.97E-02
**GO:0034645**	cellular macromolecule biosynthetic process	BP	1.02E-10
**GO:0019538**	protein metabolic process	BP	1.86E-08
**GO:0010467**	gene expression	BP	3.62E-11
**GO:0044267**	cellular protein metabolic process	BP	4.79E-10
**GO:0006412**	translation	BP	6.29E-33
**GO:0007186**	G-protein coupled receptor signaling pathway	BP	9.02E-06

**GO:0043229**	intracellular organelle	CC	1.49E-04
**GO:0005622**	intracellular	CC	3.44E-04
**GO:0044391**	ribosomal subunit	CC	4.00E-04
**GO:0005912**	adherens junction	CC	2.86E-03
**GO:0044464**	cell part	CC	2.92E-03
**GO:0044424**	intracellular part	CC	5.80E-03
**GO:0015934**	large ribosomal subunit	CC	1.70E-02
**GO:0015935**	small ribosomal subunit	CC	4.19E-02
**GO:0005840**	ribosome	CC	1.57E-35
**GO:0005737**	cytoplasm	CC	1.73E-11
**GO:0044444**	cytoplasmic part	CC	2.34E-15
**GO:0032991**	macromolecular complex	CC	3.29E-10
**GO:0043232**	intracellular non-membrane-bounded organelle	CC	6.11E-19
**GO:0004888**	transmembrane signaling receptor activity	MF	1.11E-04
**GO:0004871**	signal transducer activity	MF	1.49E-04
**GO:0004930**	G-protein coupled receptor activity	MF	1.49E-04
**GO:0045296**	cadherin binding	MF	9.17E-04
**GO:0004807**	triose-phosphate isomerase activity	MF	1.55E-02
**GO:0003735**	structural constituent of ribosome	MF	1.57E-35
**GO:0005198**	structural molecule activity	MF	4.23E-29
**GO:0004984**	olfactory receptor activity	MF	9.21E-08

Note: BP stands for biological process, CC stands for cellular component, MF stands for molecular function.

Table S10. GO term enrichment of gene families expanded in mandrill. (included in a separate excel file)

Table S11. GO and KEGG enrichment of the positively selected genes (PSGs).

GO ID	GO Term	GO Class	Adjusted P-value
GO:0016301	kinase activity	MF	6.62E-10

GO:0016772	transferase activity, transferring phosphorus-containing groups	MF	1.18E-09
GO:0016773	phosphotransferase activity, alcohol group as acceptor	MF	1.18E-09
GO:0003824	catalytic activity	MF	2.03E-09
GO:0005524	ATP binding	MF	3.42E-09
GO:0004672	protein kinase activity	MF	3.42E-09
GO:0032559	adenyl ribonucleotide binding	MF	3.80E-09
GO:0030554	adenyl nucleotide binding	MF	4.73E-09
GO:0016740	transferase activity	MF	1.76E-08
GO:0005515	protein binding	MF	3.48E-08
GO:0004713	protein tyrosine kinase activity	MF	3.64E-08
GO:0016310	phosphorylation	BP	3.85E-08
GO:0006468	protein phosphorylation	BP	7.96E-08
GO:0035639	purine ribonucleoside triphosphate binding	MF	8.16E-07
GO:0036094	small molecule binding	MF	8.22E-07
GO:0032553	ribonucleotide binding	MF	9.04E-07
GO:0032555	purine ribonucleotide binding	MF	9.04E-07
GO:0017076	purine nucleotide binding	MF	1.25E-06
GO:0000166	nucleotide binding	MF	1.40E-06
GO:0006793	phosphorus metabolic process	BP	1.24E-05
GO:0006796	phosphate-containing compound metabolic process	BP	1.24E-05
GO:0009452	RNA capping	BP	2.66E-05
GO:0007626	locomotory behavior	BP	4.05E-05
GO:0005488	binding	MF	5.19E-05
GO:0007155	cell adhesion	BP	5.68E-05
GO:0022610	biological adhesion	BP	5.68E-05
GO:0008374	O-acyltransferase activity	MF	8.68E-05
GO:0043412	macromolecule modification	BP	8.73E-05
GO:0006464	protein modification process	BP	0.000113
GO:0004525	ribonuclease III activity	MF	0.00015
GO:0000123	histone acetyltransferase complex	CC	0.000275
GO:0004252	serine-type endopeptidase activity	MF	0.000396
GO:0030507	spectrin binding	MF	0.000399
GO:0006508	proteolysis	BP	0.000485

GO:0070011	peptidase activity, acting on L-amino acid peptides	MF	0.000639
GO:0004177	aminopeptidase activity	MF	0.000665
GO:0008233	peptidase activity	MF	0.000665
GO:0046777	protein autophosphorylation	BP	0.000767
GO:0005802	trans-Golgi network	CC	0.000848
GO:0005768	endosome	CC	0.000848
GO:0005516	calmodulin binding	MF	0.001068
GO:0004842	ubiquitin-protein ligase activity	MF	0.001317
GO:0017016	Ras GTPase binding	MF	0.001317
GO:0031267	small GTPase binding	MF	0.001433
GO:0051020	GTPase binding	MF	0.001433
GO:0016881	acid-amino acid ligase activity	MF	0.002103
GO:0016747	transferase activity, transferring acyl groups other than amino-acyl groups	MF	0.003245
GO:0004175	endopeptidase activity	MF	0.003245
GO:0008236	serine-type peptidase activity	MF	0.003313
GO:0017171	serine hydrolase activity	MF	0.003313
GO:0019787	small conjugating protein ligase activity	MF	0.003335
GO:0016787	hydrolase activity	MF	0.003374
GO:0008238	exopeptidase activity	MF	0.003374
GO:0070461	SAGA-type complex	CC	0.003374
GO:0070566	adenylyltransferase activity	MF	0.003374
GO:0042558	pteridine-containing compound metabolic process	BP	0.004351
GO:0050660	flavin adenine dinucleotide binding	MF	0.004532
GO:0007610	behavior	BP	0.004535
GO:0004402	histone acetyltransferase activity	MF	0.00476
GO:0006370	mRNA capping	BP	0.005009
GO:0008174	mRNA methyltransferase activity	MF	0.005009
GO:0009057	macromolecule catabolic process	BP	0.005217
GO:0019199	transmembrane receptor protein kinase activity	MF	0.005929
GO:0015291	secondary active transmembrane transporter activity	MF	0.006141
GO:0008217	regulation of blood pressure	BP	0.006384
GO:0014706	striated muscle tissue development	BP	0.006384
GO:0060537	muscle tissue development	BP	0.006384

GO:0005887	integral to plasma membrane	CC	0.006872
GO:0031226	intrinsic to plasma membrane	CC	0.006872
GO:0051345	positive regulation of hydrolase activity	BP	0.00701
GO:0000910	cytokinesis	BP	0.008254
GO:0004568	chitinase activity	MF	0.009118
GO:0006032	chitin catabolic process	BP	0.009118
GO:0045335	phagocytic vesicle	CC	0.009118
GO:0055037	recycling endosome	CC	0.009118
GO:0030318	melanocyte differentiation	BP	0.009118
GO:0017049	GTP-Rho binding	MF	0.009118
GO:2000114	regulation of establishment of cell polarity	BP	0.009118
GO:0008344	adult locomotory behavior	BP	0.009118
GO:0043966	histone H3 acetylation	BP	0.009118
GO:0017034	Rap guanyl-nucleotide exchange factor activity	MF	0.009118
GO:0004534	5'-3' exoribonuclease activity	MF	0.009118
GO:0030914	STAGA complex	CC	0.009118
GO:0008460	dTDP-glucose 4,6-dehydratase activity	MF	0.009118
GO:0004909	interleukin-1, Type I, activating receptor activity	MF	0.009118
GO:0004334	fumarylacetoacetase activity	MF	0.009118
GO:0004349	glutamate 5-kinase activity	MF	0.009118
GO:0004350	glutamate-5-semialdehyde dehydrogenase activity	MF	0.009118
GO:0043550	regulation of lipid kinase activity	BP	0.009118
GO:0070772	PAS complex	CC	0.009118
GO:0003919	FMN adenylyltransferase activity	MF	0.009118
GO:0006747	FAD biosynthetic process	BP	0.009118
GO:0008609	alkylglycerone-phosphate synthase activity	MF	0.009118
GO:0004336	galactosylceramidase activity	MF	0.009118
GO:0006683	galactosylceramide catabolic process	BP	0.009118
GO:0008611	ether lipid biosynthetic process	BP	0.009118
GO:0016287	glycerone-phosphate O-acyltransferase activity	MF	0.009118
GO:0006516	glycoprotein catabolic process	BP	0.009118
GO:0008705	methionine synthase activity	MF	0.009118
GO:0008898	homocysteine S-methyltransferase activity	MF	0.009118
GO:0010739	positive regulation of protein kinase A signaling	BP	0.009118

	cascade		
GO:0090036	regulation of protein kinase C signaling cascade	BP	0.009118
GO:0005137	interleukin-5 receptor binding	MF	0.009118
GO:0048280	vesicle fusion with Golgi apparatus	BP	0.009118
GO:0008488	gamma-glutamyl carboxylase activity	MF	0.009118
GO:0017187	peptidyl-glutamic acid carboxylation	BP	0.009118
GO:0006348	chromatin silencing at telomere	BP	0.009118
GO:0004375	glycine dehydrogenase (decarboxylating) activity	MF	0.009118
GO:0006546	glycine catabolic process	BP	0.009118
GO:0004483	mRNA (nucleoside-2'-O-)-methyltransferase activity	MF	0.009118
GO:0080009	mRNA methylation	BP	0.009118
GO:0050902	leukocyte adhesive activation	BP	0.009118
GO:0048066	developmental pigmentation	BP	0.009118
GO:0050931	pigment cell differentiation	BP	0.009118
GO:0032878	regulation of establishment or maintenance of cell polarity	BP	0.009118
GO:0019202	amino acid kinase activity	MF	0.009118
GO:0046443	FAD metabolic process	BP	0.009118
GO:0072387	flavin adenine dinucleotide metabolic process	BP	0.009118
GO:0072388	flavin adenine dinucleotide biosynthetic process	BP	0.009118
GO:0006681	galactosylceramide metabolic process	BP	0.009118
GO:0019374	galactolipid metabolic process	BP	0.009118
GO:0019376	galactolipid catabolic process	BP	0.009118
GO:0046485	ether lipid metabolic process	BP	0.009118
GO:0016413	O-acetyltransferase activity	MF	0.009118
GO:0042084	5-methyltetrahydrofolate-dependent methyltransferase activity	MF	0.009118
GO:0070528	protein kinase C signaling cascade	BP	0.009118
GO:0018214	protein carboxylation	BP	0.009118
GO:0016642	oxidoreductase activity, acting on the CH-NH2 group of donors, disulfide as acceptor	MF	0.009118
GO:0009071	serine family amino acid catabolic process	BP	0.009118
GO:0016556	mRNA modification	BP	0.009118
GO:0045123	cellular extravasation	BP	0.009118
GO:0017137	Rab GTPase binding	MF	0.009356

GO:0006030	chitin metabolic process	BP	0.009521
GO:0016891	endoribonuclease activity, producing 5'-phosphomonoesters	MF	0.009521
GO:0015103	inorganic anion transmembrane transporter activity	MF	0.010851
GO:0007605	sensory perception of sound	BP	0.012081
GO:0003714	transcription corepressor activity	MF	0.012081
GO:0050954	sensory perception of mechanical stimulus	BP	0.012081
GO:0007067	mitosis	BP	0.016642
GO:0000280	nuclear division	BP	0.016642
GO:0044431	Golgi apparatus part	CC	0.017318
GO:0006725	cellular aromatic compound metabolic process	BP	0.017468
GO:0005452	inorganic anion exchanger activity	MF	0.018637
GO:0016055	Wnt receptor signaling pathway	BP	0.020776
GO:0070588	calcium ion transmembrane transport	BP	0.020776
GO:0004540	ribonuclease activity	MF	0.020776
GO:0000226	microtubule cytoskeleton organization	BP	0.020776
GO:0008271	secondary active sulfate transmembrane transporter activity	MF	0.020776
GO:0008272	sulfate transport	BP	0.020776
GO:0015116	sulfate transmembrane transporter activity	MF	0.020776
GO:0042813	Wnt-activated receptor activity	MF	0.020776
GO:0016573	histone acetylation	BP	0.020776
GO:0048193	Golgi vesicle transport	BP	0.020776
GO:0030574	collagen catabolic process	BP	0.020776
GO:0090382	phagosome maturation	BP	0.020776
GO:0045670	regulation of osteoclast differentiation	BP	0.020776
GO:0046920	alpha-(1->3)-fucosyltransferase activity	MF	0.020776
GO:0034450	ubiquitin-ubiquitin ligase activity	MF	0.020776
GO:0008124	4-alpha-hydroxytetrahydrobiopterin dehydratase activity	MF	0.020776
GO:0034435	cholesterol esterification	BP	0.020776
GO:0034736	cholesterol O-acyltransferase activity	MF	0.020776
GO:0006919	activation of cysteine-type endopeptidase activity involved in apoptotic process	BP	0.020776
GO:0032963	collagen metabolic process	BP	0.020776

GO:0044236	multicellular organismal metabolic process	BP	0.020776
GO:0044243	multicellular organismal catabolic process	BP	0.020776
GO:0044259	multicellular organismal macromolecule metabolic process	BP	0.020776
GO:0002761	regulation of myeloid leukocyte differentiation	BP	0.020776
GO:0030316	osteoclast differentiation	BP	0.020776
GO:0045637	regulation of myeloid cell differentiation	BP	0.020776
GO:0034433	steroid esterification	BP	0.020776
GO:0034434	sterol esterification	BP	0.020776
GO:0004772	sterol O-acyltransferase activity	MF	0.020776
GO:0010950	positive regulation of endopeptidase activity	BP	0.020776
GO:0010952	positive regulation of peptidase activity	BP	0.020776
GO:0043280	positive regulation of cysteine-type endopeptidase activity involved in apoptotic process	BP	0.020776
GO:0097202	activation of cysteine-type endopeptidase activity	BP	0.020776
GO:2001056	positive regulation of cysteine-type endopeptidase activity	BP	0.020776
GO:0008305	integrin complex	CC	0.020935
GO:0007167	enzyme linked receptor protein signaling pathway	BP	0.021323
GO:0004675	transmembrane receptor protein serine/threonine kinase activity	MF	0.022843
GO:0016050	vesicle organization	BP	0.022843
GO:0016337	cell-cell adhesion	BP	0.023783
GO:0000087	M phase of mitotic cell cycle	BP	0.023909
GO:0051301	cell division	BP	0.025184
GO:0004553	hydrolase activity, hydrolyzing O-glycosyl compounds	MF	0.025409
GO:0048037	cofactor binding	MF	0.026148
GO:0048856	anatomical structure development	BP	0.026148
GO:0030097	hemopoiesis	BP	0.026288
GO:0006475	internal protein amino acid acetylation	BP	0.026288
GO:0018393	internal peptidyl-lysine acetylation	BP	0.026288
GO:0018394	peptidyl-lysine acetylation	BP	0.026288
GO:0008237	metallopeptidase activity	MF	0.028813
GO:0048285	organelle fission	BP	0.028832

GO:0015301	anion:anion antiporter activity	MF	0.030496
GO:0043085	positive regulation of catalytic activity	BP	0.030496
GO:0001510	RNA methylation	BP	0.030496
GO:0048534	hemopoietic or lymphoid organ development	BP	0.030496
GO:0006473	protein acetylation	BP	0.030496
GO:0004521	endoribonuclease activity	MF	0.030496
GO:0004712	protein serine/threonine/tyrosine kinase activity	MF	0.030496
GO:0043473	pigmentation	BP	0.030496
GO:0017080	sodium channel regulator activity	MF	0.030496
GO:0004948	calcitonin receptor activity	MF	0.030496
GO:0046373	L-arabinose metabolic process	BP	0.030496
GO:0046556	alpha-N-arabinofuranosidase activity	MF	0.030496
GO:0004962	endothelin receptor activity	MF	0.030496
GO:0048484	enteric nervous system development	BP	0.030496
GO:0070776	MOZ/MORF histone acetyltransferase complex	CC	0.030496
GO:0042577	lipid phosphatase activity	MF	0.030496
GO:0004822	isoleucine-tRNA ligase activity	MF	0.030496
GO:0006428	isoleucyl-tRNA aminoacylation	BP	0.030496
GO:0019236	response to pheromone	BP	0.030496
GO:0080025	phosphatidylinositol-3,5-bisphosphate binding	MF	0.030496
GO:0032777	Piccolo NuA4 histone acetyltransferase complex	CC	0.030496
GO:0000103	sulfate assimilation	BP	0.030496
GO:0004020	adenylylsulfate kinase activity	MF	0.030496
GO:0004781	sulfate adenylyltransferase (ATP) activity	MF	0.030496
GO:0051018	protein kinase A binding	MF	0.030496
GO:0017025	TBP-class protein binding	MF	0.030496
GO:0034454	microtubule anchoring at centrosome	BP	0.030496
GO:0008250	oligosaccharyltransferase complex	CC	0.030496
GO:0005315	inorganic phosphate transmembrane transporter activity	MF	0.030496
GO:0034599	cellular response to oxidative stress	BP	0.030496
GO:0090307	spindle assembly involved in mitosis	BP	0.030496
GO:0004666	prostaglandin-endoperoxide synthase activity	MF	0.030496
GO:0019371	cyclooxygenase pathway	BP	0.030496

GO:0043141	ATP-dependent 5'-3' DNA helicase activity	MF	0.030496
GO:0030139	endocytic vesicle	CC	0.030496
GO:0002573	myeloid leukocyte differentiation	BP	0.030496
GO:0030010	establishment of cell polarity	BP	0.030496
GO:0030534	adult behavior	BP	0.030496
GO:0019566	arabinose metabolic process	BP	0.030496
GO:0048483	autonomic nervous system development	BP	0.030496
GO:0070775	H3 histone acetyltransferase complex	CC	0.030496
GO:0004779	sulfate adenylyltransferase activity	MF	0.030496
GO:0006677	glycosylceramide metabolic process	BP	0.030496
GO:0046477	glycosylceramide catabolic process	BP	0.030496
GO:0046514	ceramide catabolic process	BP	0.030496
GO:0046521	sphingoid catabolic process	BP	0.030496
GO:0010737	protein kinase A signaling cascade	BP	0.030496
GO:0010738	regulation of protein kinase A signaling cascade	BP	0.030496
GO:0072393	microtubule anchoring at microtubule organizing center	BP	0.030496
GO:0019369	arachidonic acid metabolic process	BP	0.030496
GO:0006342	chromatin silencing	BP	0.030496
GO:0045814	negative regulation of gene expression, epigenetic	BP	0.030496
GO:0003684	damaged DNA binding	MF	0.032579
GO:0030163	protein catabolic process	BP	0.0328
GO:0007596	blood coagulation	BP	0.03529
GO:0007599	hemostasis	BP	0.03529
GO:0006629	lipid metabolic process	BP	0.036449
GO:0016569	covalent chromatin modification	BP	0.037639
GO:0016570	histone modification	BP	0.037639
GO:0043547	positive regulation of GTPase activity	BP	0.039103
GO:0050817	coagulation	BP	0.041062
GO:0002520	immune system development	BP	0.043864
GO:0006026	aminoglycan catabolic process	BP	0.043864
GO:0004702	receptor signaling protein serine/threonine kinase activity	MF	0.043864
GO:0008235	metalloexopeptidase activity	MF	0.043864
GO:0043235	receptor complex	CC	0.043864

GO:0016407	acetyltransferase activity	MF	0.043864
GO:0043414	macromolecule methylation	BP	0.043937
GO:0048731	system development	BP	0.044356
GO:0006895	Golgi to endosome transport	BP	0.044356
GO:0000186	activation of MAPKK activity	BP	0.044356
GO:0006729	tetrahydrobiopterin biosynthetic process	BP	0.044356
GO:0030099	myeloid cell differentiation	BP	0.044356
GO:0016822	hydrolase activity, acting on acid carbon-carbon bonds	MF	0.044356
GO:0016823	hydrolase activity, acting on acid carbon-carbon bonds, in ketonic substances	MF	0.044356
GO:0046146	tetrahydrobiopterin metabolic process	BP	0.044356
GO:0006687	glycosphingolipid metabolic process	BP	0.044356
GO:0019377	glycolipid catabolic process	BP	0.044356
GO:0046479	glycosphingolipid catabolic process	BP	0.044356
GO:0046504	glycerol ether biosynthetic process	BP	0.044356
GO:0006906	vesicle fusion	BP	0.044356
GO:0043281	regulation of cysteine-type endopeptidase activity involved in apoptotic process	BP	0.044356
GO:2000116	regulation of cysteine-type endopeptidase activity	BP	0.044356
GO:0005975	carbohydrate metabolic process	BP	0.046346

Table S12. GO and KEGG enrichment of the positively selected genes related to innate immunity. (included in a separate excel file)

Table S13. Repeat content of MHC class I region for mandrill and human.

**Type**	**Mandrill**			**Human**		
	Copy Number	Length (bp)	Percent (%)	Copy Number	Length (bp)	Percent (%)
**DNA/Crypton-V**	1	65	0.00	0	0	0.00
**DNA/DNA**	3	179	0.01	2	126	0.01
**DNA/Helitron**	1	363	0.02	1	322	0.02

**DNA/Maverick**	0	0	0.00	1	44	0.00
**DNA/Sola**	0	0	0.00	1	69	0.00
**DNA/MULE-MuDR**	2	141	0.01	0	0	0.00
**DNA/TcMar-Tc1**	1	187	0.01	1	183	0.01
**DNA/TcMar-Tigge**	12	3,673	0.20	0	0	0.00
**DNA/TcMar-Tigger**	26	10,177	0.56	27	11,980	0.63
**DNA/hAT**	2	184	0.01	1	174	0.01
**DNA/hAT-Charlie**	38	9,586	0.52	46	9,941	0.52
**DNA/hAT-Tip100**	9	2,016	0.11	6	839	0.04
**LINE/CR1**	4	772	0.04	4	771	0.04
**LINE/Jockey**	0	0	0.00	1	57	0.00
**LINE/L1**	754	340,504	18.62	759	406,157	21.26
**LINE/L1-Tx1**	1	142	0.01	0	0	0.00
**LINE/L2**	38	10,351	0.57	32	8,906	0.47
**LINE/RTE-X**	2	290	0.02	2	302	0.02
**LTR/Copia**	1	92	0.01	0	0	0.00
**LTR/ERV1**	146	80,428	4.40	126	77,703	4.07
**LTR/ERVK**	22	10,368	0.57	34	29,679	1.55
**LTR/ERVL**	171	91,775	5.02	207	123,209	6.45
**LTR/ERVL-MaLR**	109	35,671	1.95	81	27,654	1.45
**LTR/Gypsy**	2	170	0.01	1	67	0.00
**LTR/LTR**	3	728	0.04	1	170	0.01
**SINE/7SL**	5	338	0.02	9	399	0.02
**SINE/Alu**	947	276,532	15.12	806	267,594	14.01
**SINE/B4**	27	1,505	0.08	24	895	0.05
**SINE/MIR**	43	5,880	0.32	44	6,508	0.34
**SINE/tRNA-7SL**	10	637	0.03	8	836	0.04
**SINE/tRNA-**	1	121	0.01	1	121	0.01

**RTE**						
**All**	2,381	882,875	48.27	2,226	974,706	51.03

Table S14. Mutation effect of some disease related genes.

PROTEIN	UNIPROT_ID	REF	ALT	POS	VAR	SIFT	Domain
**ALAD**	P13716	K	N	59	K59N	1	ALAD
**CIITA**	P33076	G	A	500	G500A	1	NACHT domain
**CRB1**	P82279	G	S	959	G959S	0.66	PFAM NO/PROSITE(EGF-like 14)
**IL4R**	J9JII2	I	V	75	I75V	0.82	Interleukin-4 receptor
**MCPH1**	Q8NEM0	A	V	761	A761V	0.52	BRCT domain
**NPHS2**	Q9NP85	I	V	192	I192V	1	SPFH domain / Band 7 family
**TP53BP1**	Q12888	D	E	353	D353E	1	not included

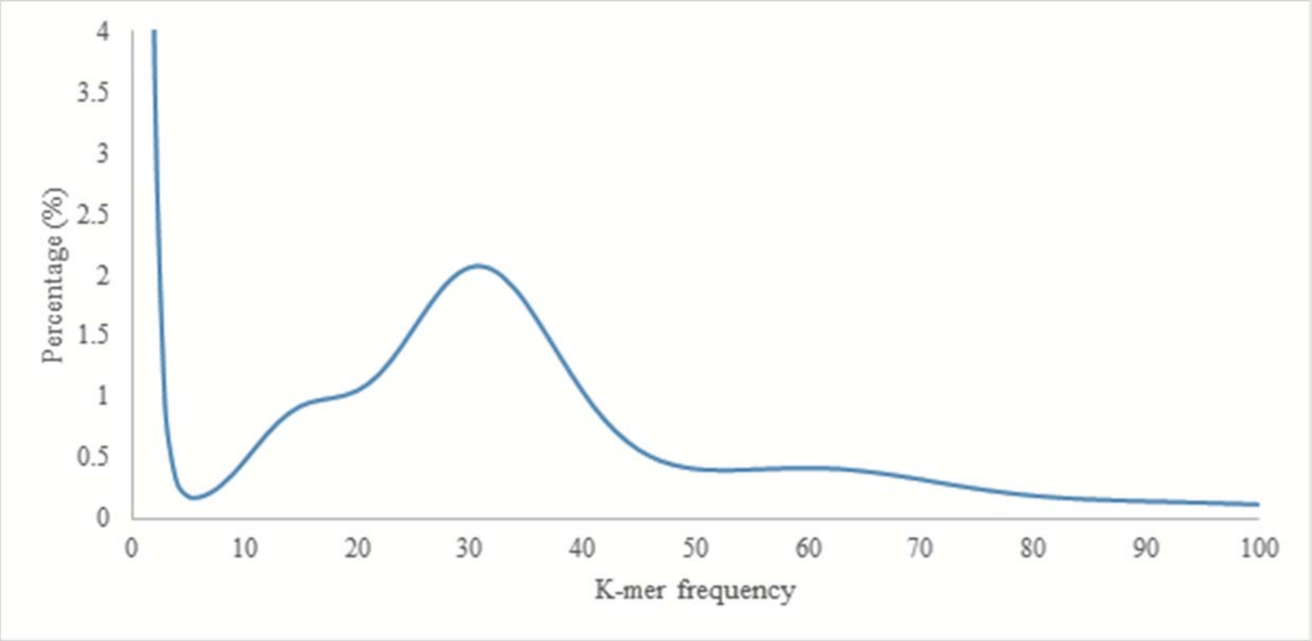

Figure S1. K-mer (k=17) distribution of the mandrill sequencing data. The x-axis is the 17mer depth (X) and the y-axis is the proportion which represents the frequency at that depth divide by the total frequency of all the depth. In the distribution of 17mer of Mandrill, it has a small peak at 1/2 the peak depth shows a slight heterozygous rate of the genome. These information for the genome has a significant guiding role at the subsequent assembled.

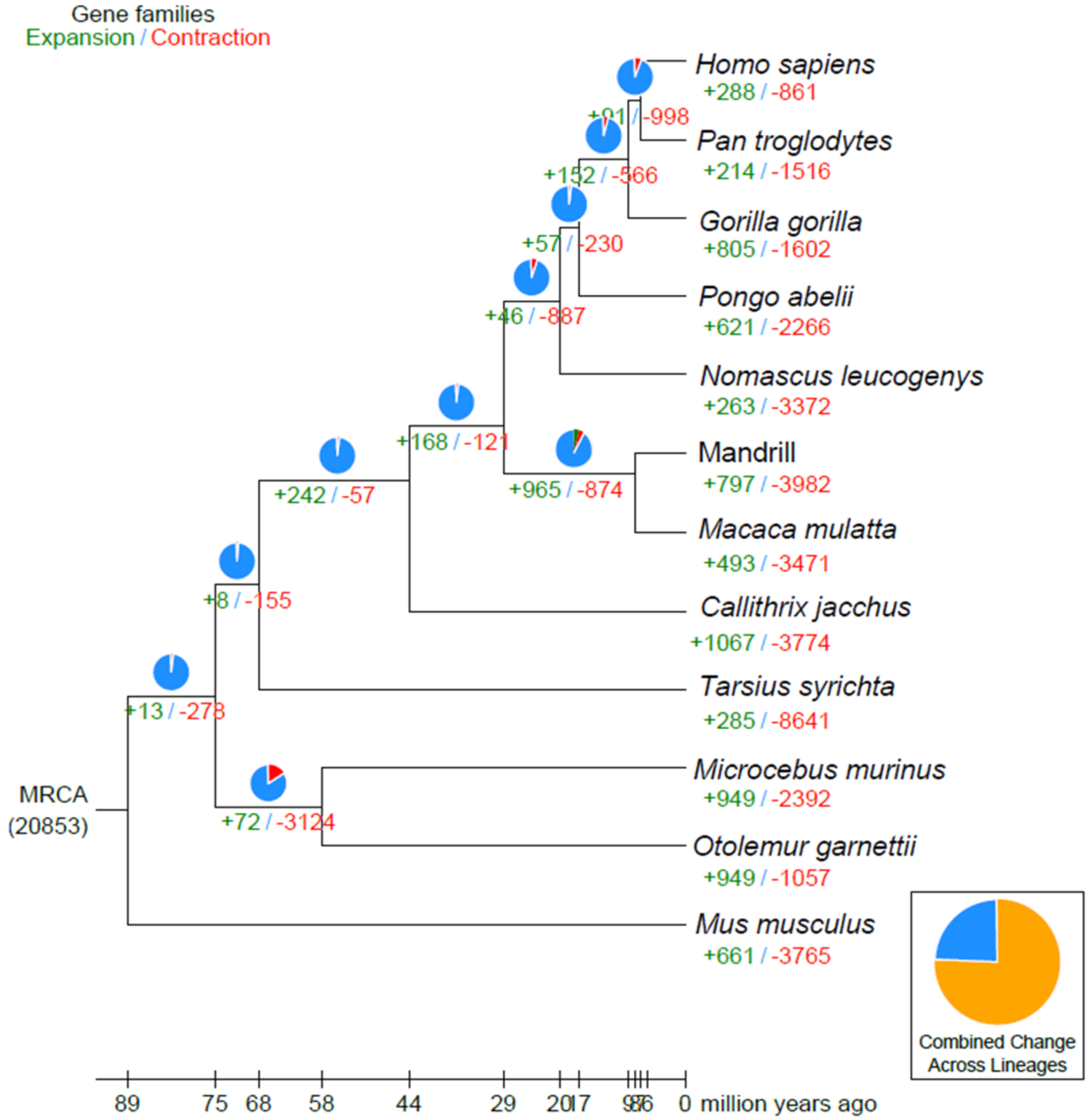

Figure S2. Phylogenetic relationships and number of gene families displaying expansion and contraction. Expansions and contractions are marked on the node.

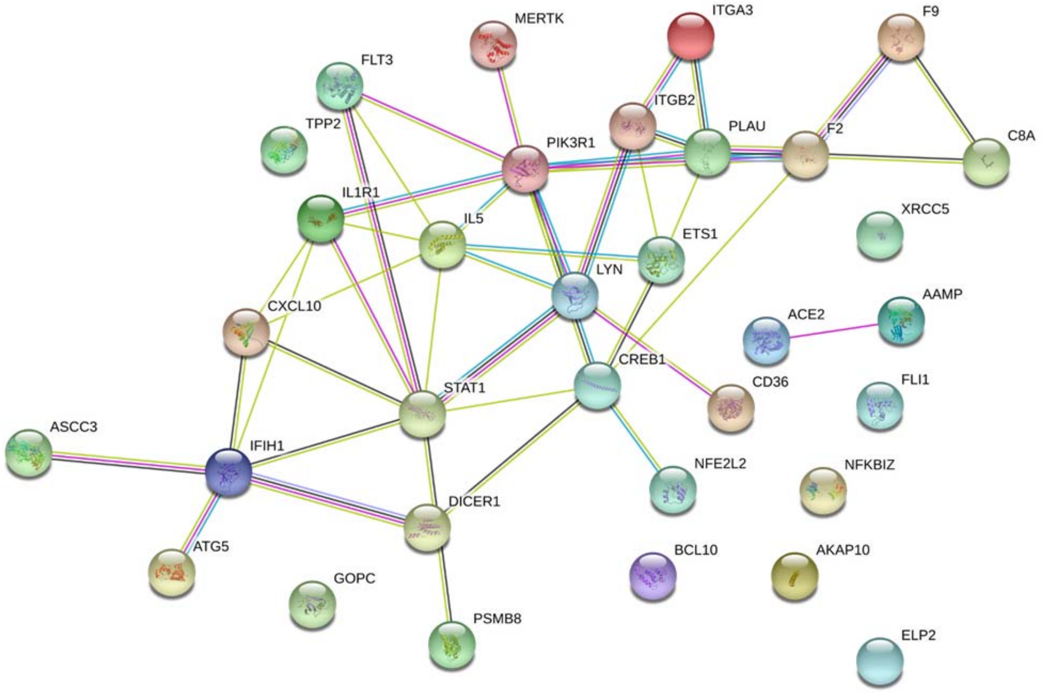

Figure S3. Interaction between innate immunity for positively selected genes in mandrill.

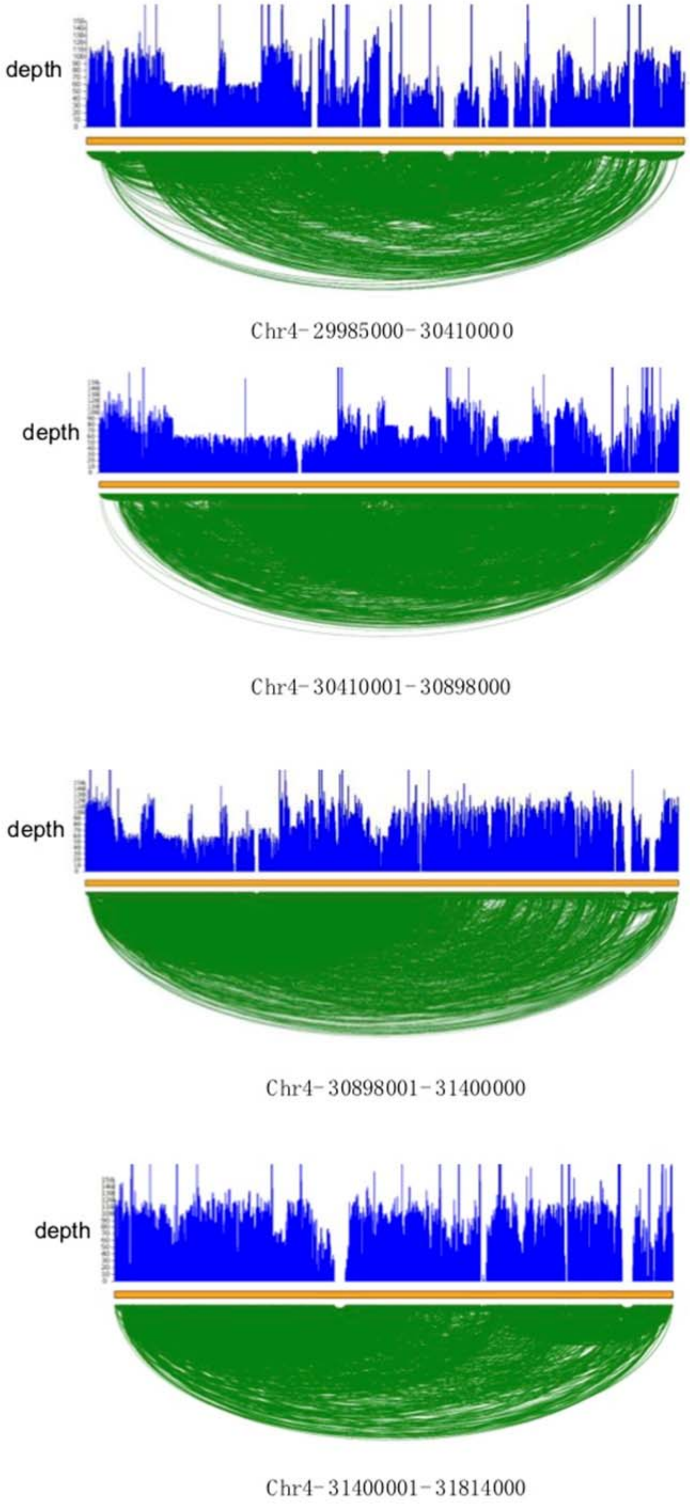

Figure S4. Sequencing depth and the location relationships of pair-end reads on MHC class I region for mandrill. Pair-end reads alignments were shown in green and coverage for each position were shown in blue.
